# High Cell-Free DNA Predicts Fatal Outcome among *Staphylococcus aureus* Bacteraemia Patients with Intensive Care Unit Treatment

**DOI:** 10.1371/journal.pone.0087741

**Published:** 2014-02-10

**Authors:** Erik Forsblom, Janne Aittoniemi, Eeva Ruotsalainen, Visa Helmijoki, Reetta Huttunen, Juulia Jylhävä, Mikko Hurme, Asko Järvinen

**Affiliations:** 1 Division of Infectious Diseases, Department of Medicine, Helsinki University Central Hospital, Helsinki, Finland; 2 Department of Clinical Microbiology, Fimlab Laboratories, Tampere, Finland; 3 Department of Microbiology and Immunology, School of Medicine, University of Tampere, Tampere, Finland; 4 Department of Internal Medicine, Tampere University Hospital, Tampere, Finland; University Hospital Medical Centre, Germany

## Abstract

**Introduction:**

Among patients with bacteraemia or sepsis the plasma cell-free DNA (cf-DNA) biomarker has prognostic value and Pitt bacteraemia scores predict outcome. We evaluated the prognostic value of plasma cf-DNA in patients with *Staphylococcus aureus* bacteraemia (SAB) treated in the ICU or in the general ward.

**Methods:**

418 adult patients with positive blood culture for *S. aureus* were prospectively followed for 90 days. SAB patients were grouped according to ICU treatment: 99 patients were treated in ICU within 7 days of documented SAB whereas 319 patients were managed outside ICU. Pitt bacteraemia scores were assessed at hospital arrival and cf-DNA was measured at days 3 and 5 from positive blood culture.

**Results:**

SAB patients with high Pitt bacteraemia scores and ICU treatment presented higher cf-DNA values as compared to SAB patients with low Pitt bacteraemia scores and non-ICU treatment at both days 3 and 5. Among ICU patients cf-DNA >1.99 µg/ml at day 3 predicted death with a sensitivity of 67% and a specificity of 77% and had an AUC in receiver operating characteristic analysis of 0.71 (p<0.01). The cut-off cf-DNA >1.99 µg/ml value demonstrated a strong association to high Pitt bacteraemia scores (≥4 points) (p<0.000). After controlling for all prognostic markers, Pitt bacteraemia scores ≥4 points at hospital admission (OR 4.47, p<0.000) and day 3 cf-DNA (OR 3.56, p<0.001) were the strongest factors significantly predicting outcome in ICU patients. cf-DNA at day 5 did not predict fatal outcome.

**Conclusion:**

High cf-DNA concentrations were observed among patients with high Pitt bacteraemia scores and ICU treatment. Pitt bacteraemia scores (≥4 points) and cf-DNA at day 3 from positive blood culture predicted death among SAB patients in ICU and were found to be independent prognostic markers. cf-DNA had no prognostic value among non-ICU patients.

## Introduction

*Staphylococcus aureus* accounts for one fourth or one fifth of all bacteraemic infections worldwide [1], [2], [3], [4]. It is among the three most common pathogens in all types of infections in critically ill patients [5]. *S. aureus* bacteraemia (SAB) is commonly found also in previously healthy individuals and it is associated with high 7–39% overall mortality [6], [7], [8]. Up to one third of SAB patients need treatment in intensive care unit (ICU) [9], [10].

Biomarkers and intensive care scoring systems have been studied as clinical tools in the evaluation of severely ill patients with bacteraemia or sepsis and they may be used as an aid in risk stratification or as a surrogate marker for patient outcome, to identify a patient with increased probability of having a disease or a pathologic process, or to follow the treatment response [11], [12], [13], [14]. Several scoring systems e.g. the Acute Physiology and Chronic Health Evaluation II (APACHE II) or SOFA score (sequential organ failure assessment) are available for assessing severity of illness and predicting outcome among ICU patients [13], [14]. The Pitt bacteraemia score system is known to reflect severity of illness among SAB patients [15], [16] and the Pitt bacteraemia scores system was recently demonstrated to better predict mortality among ICU patients with sepsis as compared to APACHE II [12].

Cell-free DNA (cf-DNA) is a biomarker that has received a lot of attention in the research of critically ill patients recently [17], [18], [19], [20], [21], [22]. The term cf-DNA implies free DNA fragments in the plasma originating from necrosis and apoptotic cells [23]. Healthy individuals display low levels of cf-DNA in plasma [24] as phagocytes remove deceased cell debris [25]. Prediction of sepsis development among both critically ill patients [17] and in hospitalized patients [20] has been reflected by elevated cf-DNA levels. Sepsis is known to enhance apoptosis [26] and cell necrosis [27] and to lead to elevated cf-DNA levels [25], [28]. Plasma cf-DNA upon ICU admission has been shown to independently correlate with serum lactate elevation among patients with severe sepsis and septic shock. This may reflect the impact of sepsis related hypoxemia on apoptosis [19]. Non-survivors in ICU have been shown to have higher cf-DNA levels than survivors [17], [18], [19], [20], [29], [30]. High cf-DNA has been shown to be an independent prognostic marker for fatal outcome among bacteraemic patients [21]. Recently, a thorough retrospective study showed a very high discriminative capability of cf-DNA to predict mortality among ICU patients with severe sepsis [30].

The capability of cf-DNA to predict infection, sepsis and mortality has been evaluated in several studies [17], [19], [20], [21], [22], [29], [30]. However, the usefulness of cf-DNA measurement in bacteraemic patients has been evaluated only in a few studies including several different bacteraemic pathogens with higher cf-DNA levels observed in ICU treated patients [21] and in non-surviving severe sepsis patients in ICU [30]. The prognostic use and cut-off values of cf-DNA regarding bacteraemic ICU patients with only one causative organism has not been studied. The aim of our study was to evaluate and compare the prognostic value of cf-DNA, and its correlation to Pitt bacteraemia scores, in SAB patients with ICU treatment and in SAB patients managed without ICU treatment. We observed that cf-DNA levels correlated significantly with Pitt bacteraemia scores and both were independent predictors for death in SAB patients requiring ICU surveillance whereas cf-DNA had no outcome predictive value among SAB patients managed without ICU treatment.

## Patient Cohort and Methods

### Ethics statement

The trial was approved by *The institutional review board of Helsinki University Central Hospital and*

*The Ethical committee of Helsinki University Central Hospital* and by each study site and a written informed consent was provided by each patient [31].

### Settings and study population

Adult patients with at least one positive blood culture for *S. aureus* were included from five university and seven central hospitals in Finland from January 1999 to May 1999 and January 2000 to August 2002. Altogether 430 SAB patients were included and followed prospectively for at least 90 days [31]. The median time between blood culture sampling and study inclusion was three days. Exclusion criteria included: age <18 years, pregnancy, breastfeeding, imprisonment, epilepsy, bacteraemia 28 days prior to the study, polymicrobial bacteraemia and meningitis [31]. All cases of methicillin-resistant *S. aureus* (MRSA) were excluded (N = 6). We documented data regarding gender, age, acquisition of SAB, underlying diseases and McCabe's classification, ICU treatment, parameters required for Pitt bacteraemia score calculation i.e. mental status, vital signs, requirement for mechanical ventilation, and recent cardiac arrest [32], acute liver of kidney failure, deep infection focus and laboratory findings including plasma cf-DNA and C-reactive protein (CRP) concentrations at days 3 and 5 from the positive blood culture sampling. Primary endpoint was regarded as mortality at 7, 28 or 90 days. Secondary end points were deep infection foci localized during the 90 days follow-up.

### Follow-up time period

None of the patients were lost during the follow-up of 90 days. Patients that were transferred to other hospitals were followed from patient records and direct contact to that hospital. Patients who were not hospitalized at 90 days had a follow-up visit at the outpatient policlinic.

### Definitions

SAB was regarded as healthcare-associated (HA) when the positive blood culture for *S. aureus* was obtained ≥48 h after hospital admission or when the patient had remained in a long-term care facility or undergone haemodialysis within the preceding two months. McCabe's criteria were used to classify underlying diseases [33]. Deep infection foci included mediastinitis, pneumonia, endocarditis, purulent arthritis, osteomyelitis, deep-seated abscess, and any foreign-body infection. Deep infection foci were documented either based on clinical suspicion or verified by bacteriological, radiological or pathological findings. Severe sepsis was classified as sepsis in combination with hypotension, hypoperfusion, or organ failure [34]. Complicated SAB was defined as SAB in combination with deep infection foci, severe sepsis, septic shock or high Pitt bacteraemia scores >4. Patients who needed ICU treatment within 7 days of positive blood culture were classified as ICU patients.

### Cell-free DNA and C-reactive protein analysis

Plasma samples for cf-DNA and CRP measurements were taken on days 3 and 5 after blood culture collection as explained above and immediately frozen to minus 70 degrees Celsius. Quant-iT™ high-sensitivity DNA assay kit and QubitH fluorometer (Invitrogen, Carlsbad, CA, USA) were used to determine cf-DNA from plasma [21]. Manufacturer's directives were followed at each laboratory step. The intra-day variation coefficients at mean cf-DNA levels of 0.734 mg/ml, 1.377 mg/ml and 4.954 mg/ml were 1.8%, 4.3% and 1.7%, respectively and the corresponding inter-day variation coefficients were 3.8%, 5.0% and 3.2%, respectively [21]. Serum or plasma C-reactive protein (CRP) (use of plasma instead of serum began 18.3.2002) was subjected to automatic immunoturbidometric analysis using analysers 917 or Modular PP-analyser (Hitachi Ltd, Tokyo, Japan) and Tina-quant CRP reagents (Roche Diagnostics, Tina-quant CRP). The normal value of CRP concentration was <10 mg/L for both methods.

### Statistical analysis

Data is presented either as absolute values and percentages or as median and interquartile ranges (IQR, 25^th^ and 75^th^ percentiles). Pearson's X^2^ -test was used to compare categorical variables whereas Mann-Whitney U-test was used for nonparametric data. Odds ratios (OR) with 95% confidence intervals (CI) were calculated. Receiver operating characteristic (ROC) curves were used to evaluate the discriminative power of cf-DNA in predicting 90-day mortality. The area under the curve (AUC) was calculated for each ROC curve. ROC-curves were drawn for cf-DNA and CRP. Univariate factors with p<0.1 were entered into a Cox regression model (proportional hazards regression) for analysis of factors predicting 90 days mortality. Analyses were done using SPSS version 12.0 (SPSS Inc., Chicago, IL, USA). All tests were two-tailed and p<0.05 was considered as significant. Youden index was defined as the sensitivity and specificity sum with the highest value or the ROC-curve point equally maximising both sensitivity and specificity values to locate the cut-off point.

## Results

### Patient characteristics and cf-DNA concentrations

Altogether 430 SAB patients were included into the study but due to missing plasma samples the results of 418 patients are shown. All patients received an effective antibiotic in vitro against their *S. aureus* blood isolate starting from the day of the positive blood culture. The majority of patients (76%) received a beta-lactam antibiotic. Vancomycin was used only in 2% of the patients and it was the only anti-microbial agent in 1% of patients.

The median cf-DNA concentrations at days 3 and 5 from the positive blood culture are shown in Table 1 stratified according to patient characteristics. At day 3, patient demographics or underlying conditions had no significant effect on the cf-DNA levels whereas at day 5, male gender (p<0.000), age over 60 years (p<0.05), alcoholism (p<0.05), diabetes (p<0.05) and coronary artery disease (p<0.01) were all associated with significantly higher cf-DNA concentrations. The lack of severe underlying diseases (i.e. McCabe's classification of healthy or nonfatal diseases) was associated to significantly lower cf-DNA values as compared to patients without these characteristics. Patients with chronic renal failure, dialysis treatment, rheumatoid or connective tissue diseases or malignancies did not present higher cf-DNA concentrations at neither 3 or at 5 days (Table 1). The cf-DNA concentrations at both days 3 and 5 correlated significantly with high Pitt bacteraemia scores (≥4 points). cf-DNA concentrations both at days 3 and 5 were significantly higher in ICU patients, in patients with a deep infection focus and in those who died as compared to patients without these factors (Table 1).

**Table 1:** Plasma cell-free DNA (cf-DNA) concentrations (µg/ml) at days 3 and 5 from the positive blood culture in 418 patients with *Staphylococcus aureus* bacteraemia stratified according to patient demographics, underlying conditions, treatment in intensive care unit and mortality.

	N = 418	Factor present^2^	Factor absent^2^	p-value^1^	Factor present^3^	Factor absent^3^	p-value^1^
**Demographics**							
Male sex	262 (63)	1.58 (1.31–1.93)	1.49 (1.26–1.91)	NS	1.52 (1.28–1.93)	1.32 (1.17–1.63)	<0.000
Age >60 years	208 (50)	1.54 (1.29–1.99)	1.55 (1.29–1.86)	NS	1.50 (1.23–1.98)	1.40 (1.21–1.77)	<0.05
Healthcare-associated	224 (54)	1.54 (1.31–1.92)	1.55 (1.27–1.92)	NS	1.46 (1.23–1.79)	1.43 (1.21–1.88)	NS
**Underlying condition**							
Healthy or nonfatal^A^	302 (72)	1.53 (1.29–1.87)	1.58 (1.34–1.99)	NS	1.39 (1.20–1.81)	1.56 (1.32–1.92)	<0.01
Alcoholism	47 (11)	1.69 (1.37–2.11)	1.52 (1.29–1.87)	NS	1.59 (1.25–2.06)	1.42 (1.21–1.80)	<0.05
Coronary artery disease	109 (26)	1.53 (1.25–1.98)	1.54 (1.30–1.89)	NS	1.62 (1.24–2.13)	1.40 (1.21–1.75)	<0.01
Diabetes with complication	105 (25)	1.64 (1.32–2.08)	1.52 (1.29–1.85)	NS	1.61 (1.27–2.01)	1.42 (1.21–1.77)	<0.05
Chronic kidney failure^B^	59 (14)	1.51 (1.29–1.71)	1.54 (1.29–1.94)	NS	1.53 (1.35–1.80)	1.41 (1.20–1.84)	NS
Dialysis (hemo or peritoneal)	46 (11)	1.51 (1.38–1.72)	1.55 (1.29–1.94)	NS	1.53 (1.35–1.78)	1.42 (1.21–1.84)	NS
Acute liver failure	5 (1)	1.46 (1.24–2.46)	1.54 (1.29–1.92)	NS	1.34 (1.19–1.98)	1.45 (1.22–1.84)	NS
Rheumatoid arthritis	24 (6)	1.43 (1.17–1.59)	1.55 (1.29–1.94)	NS	1.39 (1.15–1.62)	1.45 (1.23–1.84)	NS
Connective tissue disease	31 (7)	1.52 (1.22–2.03)	1.54 (1.29–1.91)	NS	1.40 (1.19–1.80)	1.45 (1.22–1.83)	NS
Malignancy	60 (14)	1.59 (1.32–2.02)	1.53 (1.29–1.91)	NS	1.51 (1.23–1.77)	1.43 (1.21–1.84)	NS
**PITT Bacteraemia Score**							
≥4 points (high score)	22 (5%)	2.06 (1.56–2.91)	1.53 (1.29–1.88)	<0.000	1.89 (1.59–2.61)	1.42 (1.21–1.81)	<0.001
≥3 points	29 (7%)	1.88 (1.47–2.47)	1.53 (1.28–1.87)	<0.01	1.88 (1.54–2.52)	1.42 (1.21–1.80)	<0.000
≥2 points	60 (14%)	1.67 (1.37–2.14)	1.53 (1.28–1.87)	<0.05	1.62 (1.34–1.92)	1.41 (1.21–1.81)	<0.05
**Treatment in ICU**							
At documented SAB	65 (16)	1.77 (1.49–2.27)	1.49 (1.27–1.84)	<0.000	1.77 (1.37–2.37)	1.40 (1.19–1.77)	<0.000
Within 3 days	87 (21)	1.74 (1.44–2.19)	1.49 (1.27–1.82)	<0.000	1.64 (1.32–2.26)	1.40 (1.19–1.77)	<0.000
Within 7 days	99 (24)	1.69 (1.41–2.19)	1.49 (1.26–1.81)	<0.000	1.63 (1.31–2.26)	1.40 (1.19–1.76)	<0.000
**Infection focus**							
Any deep infection^C^	349 (84)	1.56 (1.32–1.94)	1.37 (1.19–1.74)	0.001	1.49 (1.26–1.92)	1.27 (1.09–1.53)	<0.000
**Mortality**							
At 7 days	16 (4)	2.27 (1.59–3.53)	1.53 (1.29–1.88)	<0.000	1.95 (1.51–2.92)	1.42 (1.21–1.82)	<0.01
At 28 days	52 (12)	1.75 (1.44–2.52)	1.52 (1.27–1.85)	<0.000	1.72 (1.42–3.01)	1.40 (1.20–1.78)	<0.000
At 90 days	73 (18)	1.74 (1.45–2.37)	1.49 (1.27–1.83)	<0.000	1.70 (1.41–2.56)	1.39 (1.19–1.78)	<0.000

1 Mann-Whitney *U-test*.

2 At day 3 from the positive blood culture.

3 At day 5 from the positive blood culture.

A According to McCabe and Jackson [33].

B Chronically elevated serum creatinine (>180 µmol/l).

C During the 90 days follow-up.

Values are expressed as N (%), unless otherwise stated, or as median (quartiles). NS  =  non-significant.

### ICU treatment

ICU patients had significantly more often alcoholism and a deep infection focus, and their mortality was significantly higher at 7, 28 and 90 days from the documented SAB as compared to non-ICU patients (data not shown). ICU non-survivors within 7 days of documented SAB had significantly higher cf-DNA values at day 3 even when patient related factors that might affect cf-DNA concentration were looked for separately i.e. age, underlying diseases, alcoholism, severe sepsis, need for inotropic support or mechanical ventilation, reduced degree of consciousness or high Pitt bacteraemia scores (≥4 points) (Table 2). At day 5, no difference in cf-DNA between non-survivors and survivors were seen (Table 2).

**Table 2:** Plasma cell-free DNA (cf-DNA) concentrations (µg/ml) at days 3 and 5 from the positive blood culture in 99 patients with *Staphylococcus aureus* bacteraemia (SAB) treated in intensive care unit (ICU) within 7 days of documented bacteraemia.

	ICU survivors^2^	ICU non-survivors^2^	p-value^1^	ICU survivors1^3^	ICU non-survivors^3^	p-value^1^
**Background characteristics**						
Age >60 years	1.62 (1.37–2.16)	3.97 (2.55–9.46)	<0.01	1.57 (1.31–2.46)	2.27 (1.59–3.01)	NS
Healthy or nonfatal disease^A^	1.67 (1.40–2.12)	2.37 (1.80–7.92)	<0.05	1.53 (1.29–1.93)	2.27 (1.52–3.01)	NS
Alcoholism	1.68 (1.37–2.30)	5.96 (2.37–11.0)	<0.05	1.81 (1.34–1.93)	2.96 (1.70–2.73)	NS
**Disease severity at documented SAB**						
Severe sepsis	1.69 (1.40–2.11)	3.94 (1.91–7.22)	<0.05	1.64 (1.31–1.88)	2.66 (1.72–3.48)	NS
Inotropic support	1.77 (1.50–2.23)	3.05 (2.01–5.96)	<0.05	1.63 (1.29–1.97)	2.31 (1.89–3.33)	NS
Mechanical ventilation	1.82 (1.43–2.30)	3.60 (1.91–7.22)	<0.05	1.90 (1.48–2.66)	2.64 (1.71–3.48)	NS
Reduced degree of consciousness^B^	1.68 (1.37–2.38)	3.05 (2.01–5.96)	<0.05	1.76 (1.38–2.61)	2.31 (1.89–3.23)	NS
PITT Bacteraemia Score ≥4 points	1.83 (1.42–2.42)	3.05 (2.01–5.96)	<0.05	1.76 (1.45–2.45)	2.31 (1.89–3.33)	NS
Complicated SAB	1.66 (1.39–2.14)	3.07 (2.09–5.68)	<0.01	1.59 (1.29–2.12)	2.29 (1.58–3.17)	NS

1 Mann-Whitney *U-test*.

2 At day 3 from the positive blood culture.

3 At day 5 from the positive blood culture.

A According to McCabe and Jackson [33].

B Unconsciousness or somnolent.

Patients are divided according to survivors (N = 91) and non-survivors (N = 8) at 7 days follow-up. Values are given as median (quartiles). NS  =  non-significant.

### Cut-off values for cf-DNA in predicting death

**Figure 1:**
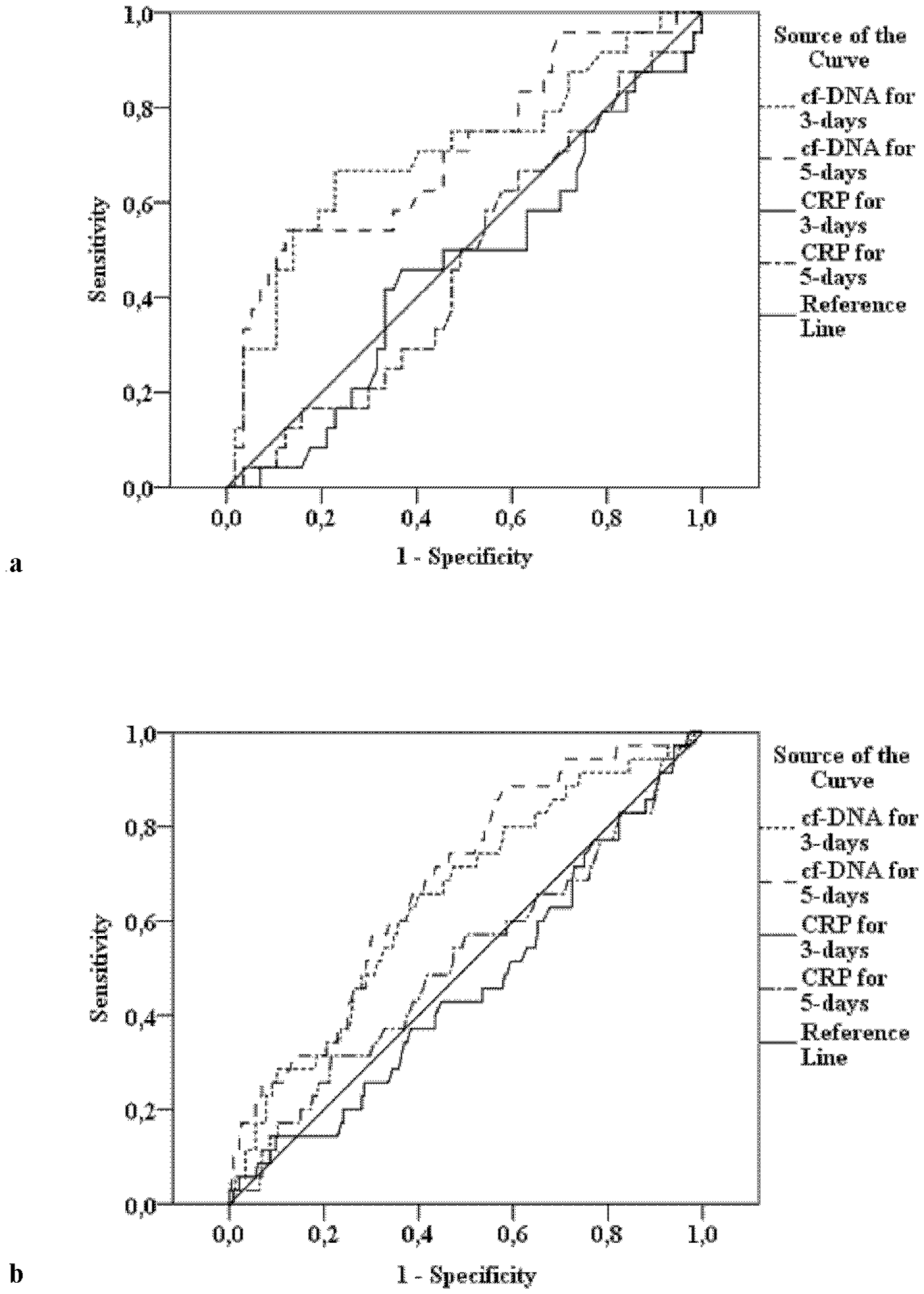
**a**: Receiver operating characteristic (ROC) curves for cf-DNA and C-reactive protein (CRP) for predicting 90-day mortality in patients with *Staphylococcus aureus* bacteraemia (SAB) treated in intensive care unit (ICU) during 7 days of documented bacteraemia (N = 99). The area under the curve (AUC) for the day 3 cf-DNA was 0.71 (95% CI 0.57–0.84) (p<0.01) with cut-off value of 1.99 µg/ml with sensitivity of 67% and specificity of 77%. For day 5 cf-DNA, the AUC was 0.71 (0.58–0.84) (p<0.01) with cut-off value of 1.69 µg/ml with sensitivity of 63% and specificity of 60%. The corresponding AUC for day 3 CRP was 0.46 (0.32–0.59) (p = 0.55) and for day 5 CRP 0.47 (0.34–0.61) (p = 0.67). **b**: Receiver operating characteristic (ROC) curves for cf-DNA and CRP for predicting 90-day mortality in patients with *Staphylococcus aureus* bacteraemia (SAB) without treatment in intensive care unit (ICU) during 7 days of documented bacteraemia (N = 319). The area under the curve (AUC) for day 3 cf-DNA was 0.64 (95% CI 0.55–0.74) (p<0.01) and cut-off value of 1.57 µg/ml with sensitivity of 62% and specificity of 61%. For day 5 cf-DNA, the AUC was 0.68 (0.59–0.77) (p<0.01) with cut-off value of 1.49 µg/ml with sensitivity of 65% and specificity of 61%. The corresponding AUC for day 3 CRP was 0.46 (0.36–0.57) (p = 0.48) and for day 5 CRP 0.51 (0.41–0.62) (p = 0.80).

In ICU patients, cf-DNA both at days 3 and 5 were found to be significant predictors for death as analysed by receiver operating characteristics (ROC) whereas CRP had no significant predictive value for mortality (Figure 1a). The AUC in the ROC analysis for the day 3 cf-DNA value in predicting death was 0.71 (95% CI 0.57–0.84, p<0.01) (Figure 1a). The cf-DNA cut-off value at day 3 was 1.99 µg/ml which predicted 90-day mortality with a sensitivity of 67% and a specificity of 77%. The cf-DNA cut-off value at day 5 was 1.69 µg/ml with a sensitivity of 63% and a specificity of 60% and an AUC of 0.71 (95% CI 0.58–0.84, p<0.01) in predicting death during 90 days follow-up (Figure 1a). When the ROC-curve analyses were repeated by grouping SAB patients according to ICU (N = 87) and non-ICU (N = 331) treatment within the first 3 days instead of the first 7 days the results were almost identical (data not shown). In non-ICU patients, the cf-DNA cut-off value at day 3 was 1.57 µg/ml which predicted 90-day mortality with a sensitivity of 62% and a specificity of 61%, and the AUC of 0.64 (95% CI 0.55–0.74, p<0.01) (Figure 1b). The cf-DNA cut-off value at day 5 was 1.49 µg/ml with a sensitivity of 65% and a specificity of 61%, and the AUC was 0.68 (95% CI 0.59–0.77, p<0.01) in predicting death for 90 days follow-up.

The patient population was stratified according to the day 3 cf-DNA cut-off values of 1.99 µg/ml (ICU patients) and 1.57 µg/ml (non-ICU patients), respectively (Table 3). Factors significantly associated to higher cf-DNA (>1.99 µg/ml) were septic shock (p<0.01), mechanical ventilation (p<0.000), reduced consciousness (p<0.01), complicated SAB (p<0.01) as well as high Pitt bacteraemia score (p<0.000). Moreover, a fatal outcome, irrespective of death time, was significantly associated to high cf-DNA values (p<0.000). However, once again, acute or chronic renal failure, dialysis, acute or chronic liver failure, malignancies or deep infection foci had no significant association with the higher cf-DNA (Table 3).

**Table 3:** Underlying conditions, severity of illness and mortality in 418 patients with *Staphylococcus aureus* bacteraemia stratified according to the cf-DNA cut-off value at day 3 for ICU (1.99 µg/ml) and non-ICU (1.57 µg/ml) patients.

	>1.99^1^	<1.99^2^	OR (95% CI)	p-value	>1.57^3^	<1.57^4^	OR (95% CI	p-value
**Background characteristics**								
Male sex	52 (60)	210 (63)	0.89 (0.55–1.45)	NS	126 (68)	136 (58)	1.52 (1.02–2.28)	<0.05
Healthy or nonfatal^A^	45 (52)	179 (54)	0.94 (0.58–1.51)	NS	127 (68)	175 (75)	0.73 (0.47–1.12)	NS
Coronary artery disease	25 (29)	84 (25)	1.21 (0.71–2.05)	NS	49 (26)	60 (26)	1.04 (0.67–1.61)	NS
Chronic renal failure^B^	9 (10)	50 (15)	0.66 (0.31–1.40)	NS	25 (13)	34 (14)	0.92 (0.52–1.59)	NS
Chronic liver failure	8 (9)	57 (17)	0.49 (0.23–1.08)	NS	22 (12)	43 (18)	0.59 (0.33–1.04)	NS
Connective tissue disease^C^	10 (11)	38 (11)	1.01 (0.49–2.14)	NS	17 (9)	31 (13)	0.66 (0.35–1.23)	NS
Malignancy	14 (16)	46 (14)	1.21 (0.63–2.32)	NS	29 (15)	31 (13)	1.21 (0.70–2.09)	NS
**Severity of illness**								
SIRS-Sepsis	54 (63)	177 (53)	1.48 (0.91–2.41)	NS	114 (61)	117 (50)	1.59 (1.08–2.36)	NS
Severe sepsis	2 (2)	13 (4)	0.58 (0.13–2.54)	NS	8 (4)	7 (3)	1.46 (0.52–4.10)	NS
Septic shock	7 (8)	5 (2)	5.79 (1.79–18.7)	<0.01	10 (5)	2 (1)	6.60 (1.43–30.5)	<0.01
Mechanical ventilation	9 (10)	7 (2)	5.43 (1.96–15.0)	<0.000	11 (5)	5 (2)	2.88 (0.98–8.45)	<0.05
Reduced consciousness^D^	10 (11)	14 (4)	2.99 (1.28–6.98)	<0.01	12 (6)	12 (5)	1.28 (0.56–2.91)	NS
Acute dialysis need	1 (1)	4 (1)	0.97 (0.11–8.74)	NS	3 (1)	2 (1)	1.90 (0.32–11.5)	NS
Acute liver failure	2 (2)	3 (1)	2.61 (0.43–15.9)	NS	2 (1)	3 (1)	0.84 (0.14–5.07)	NS
ALAT elevation ×2^E^	4 (4)	12 (4)	1.08 (0.33–3.55)	NS	7 (4)	9 (4)	0.92 (0.33–2.58)	NS
Complicated SAB	70 (81)	283 (85)	5.03 (1.53–16.5)	<0.01	164 (88)	189 (81)	1.78 (1.01–3.12)	<0.05
**PITT Bacteraemia Score**								
≥4 points (high score)	11 (12)	11 (3)	4.28 (1.79–10.2)	<0.000	15 (8)	7 (3)	2.85 (1.14–7.14)	<0.05
≥3 points	12 (14)	17 (5)	3.00 (1.38–6.56)	<0.01	18 (10)	11 (5)	2.18 (1.00–4.73)	<0.05
≥2 points	18 (21)	42 (12)	1.83 (0.99–3.37)	<0.05	33 (18)	27 (11)	1.66 (0.96–2.87)	NS
**Deep infection foci** F								
Any deep infection foci	75 (87)	274 (83)	1.44 (0.72–2.89)	NS	161 (87)	188 (80)	1.61 (0.94–2.75)	NS
**Mortality**								
Died within 7 days	10 (11)	6 (2)	7.15 (2.52–20.3)	<0.000	3 (1)	13 (5)	5.79 (1.63–20.7)	<0.01
Died within 28 days	23 (26)	29 (9)	4.81 (2.07–7.06)	<0.000	34 (18)	18 (8)	2.69 (1.46–4.94)	<0.01
Died within 90 days	31 (36)	42 (12)	3.89 (2.25–6.72)	<0.000	49 (26)	24 (10)	3.14 (1.84–5.35)	<0.000

1 N = 86,

2 N = 332,

3 N = 185,

4 N = 233.

A According to McCabe and Jackson [33].

B Chronically elevated creatinine (>180 µmol/l).

C Includes rheumatoid arthritis.

D Unconsciousness or somnolent.

E Serum Alanine-aminotransferase elevation >2 times general reference.

F Infection foci during 90 days follow-up.

Values are expressed as N (%). OR  =  odds ratio (95% confidence intervals) NS  =  non-significant.

For patients receiving ICU treatment within 7 days of documented SAB, the factors in univariate analysis associated to 90-day mortality in were age >60 years (OR 3.64, p<0.01), lack of fatal underlying diseases (OR 0.33, p<0.05), inotropic support (OR 3.19, p<0.05), mechanical ventilation (OR 2.67, p<0.05), Pitt bacteraemia scores (≥4 points) (OR 3.19, p<0.01) and cf-DNA >1.99 µg/ml at day 3 (OR 5.24, p<0.000). In multivariate analysis, only lack of fatal underlying diseases (OR 0.34, CI 95% 0.15–0.77, p<0.05), cf-DNA >1.99 µg/ml at day 3 (OR 3.56, CI 95% 1.69–7.59, p<0.001) and Pitt bacteraemia score ≥4 points (OR 4.47, 95% CI 1.94–10.3, p<0.000) were observed to be significantly associated to mortality (Table 4).

**Table 4:** Prognostic factors for 90 days mortality in patients with *Staphylococcus aureus* bacteraemia stratified according to intensive care unit treatment within 7 days of documented bacteraemia.

	Univariate analysis OR (95% CI)	p-value	Multivariate analysis OR (95% CI)	p-value
**Intensive care unit patients (N = 99)**				
Age >60 years	3.64 (1.43–9.29)	<0.01	—	—
Healthy or nonfatal^A^	0.33 (0.13–0.81)	<0.05	0.34 (0.15–0.77)	<0.05
Corticosteroid use^B^	3.21 (0.67–15.3)	NS	—	—
Chronic renal failure^C^	0.71 (0.14–3.75)	NS	—	—
Inotropia need	3.19 (1.18–8.64)	<0.05	—	—
Mechanical ventilation	2.67 (1-04–6.86)	<0.05	—	—
PITT Bacteraemia Score ≥4	3.19 (1.18–8.64)	<0.01	4.47 (1.94–10.3)	<0.000
cf-DNA cut-off 1.99 µg/ml^D^	5.24 (2.03–13.5)	<0.000	3.56 (1.69–7.59)	<0.001
**Non intensive care unit patients (N = 319)**				
Age >60 years	3.04 (1.49–6.18)	<0.01	—	—
Healthy or nonfatal^A^	0.13 (0.06–0.26)	<0.000	0.26 (0.11–0.63)	<0.01
Corticosteroid use^B^	7.91 (3.66–17.1)	<0.000	2.89 (1.39–6.07)	<0.01
Dialysis treatment	3.70 (1.70–8.07)	<0.01	—	—
Haematological malignancy	3.54 (1.02–12.3)	<0.05	—	—
Chronic lung disease	3.17 (1.58–6.39)	<0.01	2.45 (1.21–4.96)	<0.05
Any deep infection	3.67 (1.09–12.3)	<0.05	—	—
cf-DNA cut-off 1.57 µg/ml^D^	2.86 (1.45–5.64)	<0.01	—	—

A According to McCabe and Jackson [33].

B Systemic prednisone >10 mg/day or equivalent for over 1 month.

C Chronically elevated creatinine (>180 µmol/l).

D The cut-off value at day 3 according to Figure 1a.

Data is shown as odds ratios (OR) (95% confidence intervals). NS  =  non-significant.

For non-ICU patients, all Table 4 listed univariate parameters were associated significantly to 90-day mortality whereas in multivariate analysis lack of fatal underlying diseases (OR 0.26, p<0.01), corticosteroid use (OR 2.89, p<0.01) and chronic lung disease (OR 2.45, p<0.05) significantly predicted 90-day outcome. Among non-ICU patients the cf-DNA cut-off value had no prognostic impact in multivariate analysis (Table 4). When the cut-off values for days 3 and 5 in ICU patients were analysed together in multivariate analysis only the day 3 cf-DNA cut-off value significantly predicted death (data not shown). Similar results were obtained when ICU treatment during the first 3 days only were analysed (data not shown).

## Discussion

The main finding of the present study was that plasma cf-DNA concentrations were higher in SAB patients with high Pitt bacteraemia scores and ICU treatment as compared to non-ICU patients. In addition, cf-DNA levels both at days 3 and 5 were significantly higher in ICU patients with fatal outcome as compared to survivors irrespective of the death time i.e. if it occurred during the first week, 28 days or 90 days. The strongest predictors for a fatal outcome among ICU patients were cf-DNA at day 3 and Pitt bacteraemia scores ≥4 points. However, at day 5, cf-DNA concentration depended more on patient age and underlying diseases and when they were taken into account in multivariate analysis cf-DNA at day 5 was not a significant prognostic marker. To our knowledge, this is the first study where the prognostic value of cf-DNA of patients with same condition in ICU and outside it has been compared.

Higher cf-DNA values among critically ill non-survivors as compared to surviving patients have been reported earlier in many conditions [17], [18], [19], [20], [29], [30]. Five studies including 52 to 255 patients with fever of unknown origin, infection, sepsis, severe sepsis, septic shock or other ICU patients determined cf-DNA at admission, at 19 h and at 72 h after admission [17], [19], [20], [29], [30]. cf-DNA predicted the presence of infection among febrile patients with AUC of 0.99 with 95% sensitivity and 96% specificity and sepsis with AUC of 0.95 and 77% sensitivity and 94% specificity [20]. For prediction of fatal outcome, the sensitivity of cf-DNA has ranged from 60% to 92% and the specificity from 67% to 80% during 0 to 72 hours from ICU admission. [17], [19], [29]. AUC values in ROC analysis for fatal outcome in these studies has ranged from 0.70 to 0.88. Recently, cf-DNA determined at ICU admission predicted mortality among ICU patients with severe sepsis with high sensitivity of 87% and specificity of 93% and an AUC of 0.97 [30]. Clearly lower predictive cf-DNA values were observed in a larger study including 580 critically ill patients receiving mechanical ventilation with cf-DNA determination at ICU admission and at day 2 [22]. The predictive value for fatal outcome in our study had an AUC of 0.71, sensitivity of 67% and specificity of 77% which were lower than the higher end of previously reported values but comparable to those seen in studies with more variable patient cohorts [17], [19], [20], [21], [22], [29]. However, the clinical usefulness of cf-DNA determinations is still complicated by the use of two different cf-DNA measurement scales. When cf-DNA is measured straight from plasma the micro- or nanogram per millilitres (µg/ml or ng/ml) [17], [21] scale is used whereas when cf-DNA quantification is done with qPCR the results are commonly given as genome equivalents per millilitres (GE/ml) [19], [20], [22], [29].

The present study differed from the previous studies in several aspects which certainly has had an impact in lowering the specificity of the prognostic value of cf-DNA and thus may complicate the comparison of our results to many previous studies. Firstly, cf-DNA was measured in relation to documented bacteraemia and not in relation to ICU admission as in the previous studies [17], [19], [22], [29], [30]. Secondly, cf-DNA was measured also later i.e. at day 5 as compared to previous studies where the last measurement was made at 72 hours from ICU admission. Thirdly, we used Pitt bacteraemia scores and we did not determine Acute Physiology and Chronic Health Evaluation II (APACHE II) scores [13] or sequential organ failure assessment (SOFA) scores [14] which have been included in most studies evaluating the prognostic value of cf-DNA among ICU patients [17], [18], [19], [20], [21], [22], [29], [30]. However, Pitt bacteraemia scoring has been demonstrated to predict mortality among ICU sepsis patients more accurately as compared to APACHE II [12] and Pitt bacteraemia scores have previously been applied in patients with SAB [15], [16].

The present study demonstrated strong significant association between cf-DNA, Pitt bacteraemia scores and outcome among ICU patients. High Pitt bacteraemia scores correlated with high cf-DNA and with fatal outcome. The time point of cf-DNA measurements were not correlated to clinical deterioration i.e. ICU admission but to a fixed time point of the disease which is a novelty of this study but has certainly led to lower cf-DNA concentrations in patients who deteriorated later. Even without ICU risk scores it is evident that a part of most severely ill patients have been missed in the present study since we only had 4% of ICU patients classified as severe sepsis whereas in another previous study with highest specificity and sensitivity for the prognostic values of cf-DNA they had 100% of patients with severe sepsis and APACHE points over 20 [30]. The low 12% mortality within 28 days in our patient cohort was at the lower level as compared to previously published in SAB and also confirms that some severely ill patients have been missed [6], [7], [8]. However, in our patient cohort 24% of patients needed ICU treatment which corresponds well with the proportion described in other studies of 11-32% [9], [35], [36]. Out of ICU patients, 8% deceased within one week of bacteraemia. This mortality rate was far lower than those in earlier studies of 25–34% [17], [29] whereas another former Finnish study had low ICU mortality rate of 13% [19]. All these facts aim to lower the power of the present study and still cf-DNA was observed to be a significant prognostic marker specifically in SAB patients treated in ICU.

When all prognostic markers were accounted for, Pitt bacteraemia scores ≥4 points and day 3 cf-DNA cut-off value were the strongest factors predicting fatal outcome among the ICU patients with McCabes' healthy-nonfatal classification being the third parameter with significant prognostic impact. The same result was achieved when ICU treatment within the first 3 or 7 days were looked for. This is in line with data from the study where only patients with high (>20) APACHE scores were included [30]. Male gender and alcoholism have earlier been connected to higher baseline cf-DNA values [21] whereas some studies have found no connection between cf-DNA and age or gender [18], [29]. Among ICU patients, non-survivors had significantly higher cf-DNA at day 3 whereas non-significant difference was seen at day 5. This finding suggests that early apoptosis in SAB in ICU treated patients might be one of the factors leading to fatal outcome.

We included only bacteraemia due to *S. aureus* which reduces the number of possible variables. Due to low prevalence of MRSA in Finland all patients received effective antimicrobial therapy and it was instituted to all patients on the day when the positive blood culture was drawn. This also reduced one factor difficult to control when evaluating the usefulness of a new biomarker. Delayed effective antimicrobial therapy has been reported to be one of the main risk factors for poor prognosis [37]. MRSA is associated with poor prognosis and delay in effective antibiotic therapy [6], [38], [39]. Vancomycin treatment is reported to result in higher risk for recurrence and persistence of SAB when compared to treatment with the staphylococcal penicillin cloxacillin [40]. The well controlled prognostic factors made it possible to observe variables that affect cf-DNA levels.

We observed that patients in ICU had clearly higher cf-DNA levels as compared to non-ICU patients which could be expected as cf-DNA values have generally been reported to correlate well with the SOFA and APACHE scores in critically ill patients [17], [18], [19], [20], [30]. Also in the present study, a strong correlation between high cf-DNA levels and high Pitt bacteraemia scores was seen. In a previous study with only bacteraemic patients with various pathogens (*S. aureus*, *Streptococcus pneumoniae*, β-hemolytic streptococcae or *Escherichia coli*) higher cf-DNA values in ICU treated patients was reported but the cut-off values were not studied separately [21]. In the present study the difference in cf-DNA cut-off value between ICU and non-ICU was more evident at day 3 as compared to day 5. Furthermore, in non-ICU patients, cf-DNA was not an independent predicting factor for mortality suggesting that cf-DNA might have its best clinical use in predicting outcome early among ICU patients.

In the present study, acute or chronic renal or liver failure or elevated alanine-aminotransferase liver values were not associated to higher cf-DNA concentrations. Experimental animal studies indicate that liver and kidneys are responsible for cf-DNA clearance although the exact clearance process is unknown [41]. One study reported higher cf-DNA levels among critically ill patients with acute renal failure requiring renal support [17] whereas in another study no difference in DNA concentrations between healthy controls and predialysis patients was observed [42]. The exact clearance mechanism for cf-DNA requires further investigations and currently the impact of acute or chronic liver or renal failure on cf-DNA levels remains to be established.

Along with the cell-free DNA as a biomarker, procalcitonin and interleukin-10 have been investigated as promising biomarkers for patients with bacteraemia or sepsis. Recently, procalcitonin was presented as a predictor for endocarditis in SAB [43] and proposed to be a superior predictor for sepsis [20], [44]. To the best of our knowledge, no reports have investigated the prognostic value of procalcitonin solely in SAB patients. Interleukin-10 was recently demonstrated to be an independent mortality predictor in SAB patients with survivors having normal interleukin-10 levels [45] but interleukin-10 has been demonstrated to be a weaker predictor of ICU mortality among sepsis patients as compared to cf-DNA [30]. Further investigations are required to determine the relationship between cf-DNA, procalcitonin and interleukin-10 as biomarkers for bacteraemia and sepsis patients.

In conclusion, this study is the first one to demonstrate that cf-DNA levels significantly correlated with higher Pitt bacteraemia scores and ICU treatment among SAB patients as compared to non-ICU SAB patients. Among ICU patients, when all prognostic markers were accounted for, high Pitt bacteraemia scores and day 3 cf-DNA cut-off value were the strongest factors significantly predicting death. cf-DNA had no prognostic value among non-ICU SAB patients.

### Key messages

- Plasma cell-free DNA correlated with high Pitt bacteraemia score and was higher in SAB patients treated in ICU as compared to non-ICU patients.
- Plasma cell-free DNA at day 3 and 5 were significantly higher in ICU patients with fatal outcome irrespective of death time.
- High Pitt bacteraemia scores (≥4 points) and day 3 plasma cell-free DNA were the only factors that significantly predicted outcome in SAB patients with ICU treatment.
